# Disseminated Blastomycosis Presenting as Oligoarthritis, Pneumonia, and Skin Disease in an Immunocompetent Child

**DOI:** 10.1155/2021/3872354

**Published:** 2021-09-16

**Authors:** Emily Schildt, Robyn Bockrath

**Affiliations:** ^1^Ann & Robert H. Lurie Children's Hospital of Chicago, Chicago, IL, USA; ^2^Northwestern University Feinberg School of Medicine, Chicago, IL, USA

## Abstract

*Blastomyces* species (spp) can cause clinical disease affecting nearly every organ system, including the skeletal system. However, isolated joint involvement without concurrent osteomyelitis is rare, especially in children. We present a pediatric case from a tertiary care center in urban Chicago of disseminated blastomycosis caused by *Blastomyces dermatitidis* presenting as oligoarthritis (in the absence of osteomyelitis), pneumonia, and skin involvement, with clinical improvement on IV amphotericin and oral azole treatment.

## 1. Introduction

Blastomycosis is a rare endemic fungal infection, primarily found in the eastern half of the United States and Canada [1], caused by the dimorphic fungus *Blastomyces* spp. It predominantly affects adults, with only 5–10% of cases occurring in the pediatric population [2, 3]. Blastomycosis typically causes pulmonary disease first, but disseminated extrapulmonary infection can affect nearly every organ [4]. Studies have shown 21–46% of children with blastomycosis develop extrapulmonary symptoms, most classically involving the skin and skeletal systems [2, 3]. Bony involvement typically manifests as osteomyelitis [5, 6]; arthritis is much less commonly associated with *Blastomyces* spp and is thought to occur from direct extension from bone to nearby joints, resulting in septic arthritis [7].

There have been a few case reports and studies describing septic arthritis caused by blastomycosis in adults [8–15]. However, to our knowledge, this is the first reported pediatric case of disseminated blastomycosis presenting as oligoarthritis (without concurrent osteomyelitis) in association with pneumonia and skin disease in an immunocompetent child.

## 2. Case Presentation

A previously healthy 14-year-old boy was admitted to a hospital in urban Chicago for 24 hours of monitoring due to cough, tachypnea, and intermittent hypoxemia. He had a lobar infiltrate on chest X-ray and was diagnosed with presumed atypical pneumonia and discharged home to complete a 7-day course of amoxicillin and a 5-day course of azithromycin. However, he continued to experience cough and intermittent shortness of breath, even after completion of antibiotic courses.

One week after discharge, he developed stiffness, pain, and swelling of his left knee with difficulty ambulating. He was seen in the Emergency Department after symptoms had been present for approximately 6 days, at which time he had a bedside arthrocentesis performed by orthopedic surgery for presumed septic arthritis of the left knee. Synovial fluid analysis was significant for >200,000 red blood cells/*µ*L and >4,000 white blood cells/*µ*L with neutrophilic predominance, and serum studies revealed leukocytosis and elevated inflammatory markers. Magnetic resonance imaging (MRI) of the left knee was obtained without evidence of osteomyelitis. He was started on empiric antibiotics and admitted to the general pediatrics service. History was pertinent for no recent travel outside of Chicago, no camping or hiking, and no tick bites.

After admission, the patient developed daily fevers, despite having been reportedly afebrile at home. Bacterial blood cultures were obtained. He was taken to the operating room (OR) for a washout of his knee two days after admission due to persistent fevers, knee swelling, and elevated inflammatory markers. At that time, bacterial, viral, and fungal workup was sent from the synovial fluid. He had no growth from bacterial blood cultures or synovial fluid cultures. Fungal blood culture was not sent, but urine and serum *Histoplasma* and *Blastomyces* spp antibody and antigen testing was obtained.

Urine and serum *Histoplasma* and *Blastomyces* spp antigens resulted as positive, fungal culture from the synovial fluid grew *Blastomyces dermatitidis*, and 18S ribosomal DNA (rDNA) sequencing from the synovial fluid confirmed the presence of *Blastomyces* spp with no evidence of *Histoplasma*. This is indicative of likely cross-reactivity leading to false positive *Histoplasma* result from serum and urine antigen testing. All other studies including potassium hydroxide (KOH) prep, mycobacterium culture, and 16S rDNA sequencing from the synovial fluid were negative.

Of note, this patient's serum *Blastomyces* spp antibody resulted as negative, although in discussion with our infectious disease specialist and review of the literature, lack of antibody positivity is relatively common during acute infection [2].

While awaiting the results of fungal testing, the patient had been continued on empiric antibiotics and was persistently febrile with no improvement in the fever curve. Once the diagnosis was made, he was switched from antibiotics to IV amphotericin for 10 days. He was then transitioned to itraconazole due to acute kidney injury (AKI) attributed to amphotericin. After starting appropriate antifungal therapy, he had gradual improvement in fever curve, knee swelling, and inflammatory markers.

**Figure 1:**
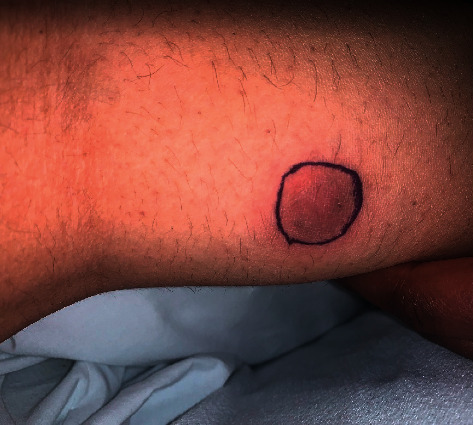
Violaceous skin nodule on posterior right lower leg, outlined with surgical marker to track size.

Clinical course was complicated by worsened hypoxemic respiratory distress in the setting of AKI and fluid overload, requiring brief pediatric intensive care unit (PICU) transfer for closer monitoring. Additionally during hospitalization, a 2 cm, raised, violaceous skin nodule on the right leg was identified, thought to be consistent with a blastomycosis skin lesion (Figure 1). No biopsy was performed given its clinical consistency with the patient's diagnosis.

At the time of publication, he is doing well on itraconazole with close outpatient follow-up in the infectious disease clinic and is regaining ambulation and movement of his affected knee with physical therapy.

## 3. Discussion

In this patient, the combination of recent pneumonia and new onset joint swelling was unusual and prompted a more extensive evaluation to diagnostically connect pulmonary and articular involvement. His inconclusive imaging and laboratory evaluation as well as lack of clinical improvement on antibiotics while awaiting culture and fungal testing results was also concerning and pointed away from a classic bacterial septic arthritis. The development of a nodular skin lesion further raised suspicion for a systemic fungal etiology. Of note, 18S and 16S rDNA gene sequencing studies were sent as part of our workup to evaluate more closely for fungal and bacterial identification, respectively, as our culture came from a patient pretreated with antibiotic therapy.

Blastomycosis is more common in immunocompromised patients, with one review of adult patients showing that only 12% of patients with blastomycosis were immunocompetent [16]. Interestingly, while studies have shown that immunocompromised patients are at higher risk for severe disease, they have similar rates of dissemination as immunocompetent patients [16, 17].

There are only a few isolated reported cases of septic arthritis as the presenting symptom of blastomycosis in children [18, 19], but this is typically in the setting of concurrent osteomyelitis due to the proposed mechanism of joint involvement being secondary to direct bony spread from an affected limb [7]. One case series described 7 children with disseminated blastomycosis causing osteomyelitis with extension to the joint [20], but none of these children had isolated arthritis. Therefore, our patient's presentation of oligoarthritis without osteomyelitis is very unusual and has not been reported in the literature surrounding blastomycosis in children.

Treatment guidelines for blastomycosis in children generally recommend amphotericin B for one to two weeks, following by 6–12 months of itraconazole therapy, with 12 months recommended for patients with osteoarticular disease like the patient we have described. Itraconazole dosing is then adjusted based on serum trough measurements to ensure adequate drug levels. Patients with central nervous system involvement may require longer courses of amphotericin B, followed by treatment with an azole for at least 12 months [21].

Although fungal infections are generally rare in the pediatric population, especially in immunocompetent children, it is important to have a high level of suspicion for the evaluation of fungal etiologies such as *Blastomyces* spp when practicing in an endemic area (such as the case for our patient, who lives in a state adjacent to the Great Lakes which has a relatively high incidence of *Blastomyces* spp [21]) or treating patients who have traveled to an area with fungal pathogens. Blastomycosis can be associated with significant morbidity and mortality, particularly when the diagnosis is delayed [2], highlighting the importance of maintaining a broad differential diagnosis, particularly when a patient does not demonstrate clinical improvement with appropriate antibiotic therapy or when multiple organ systems are involved.

## Data Availability

If you would like to access further data from this case report, please contact the corresponding author at eschildt@luriechildrens.org. No datasets were used for this paper.
